# Ideological resistance to veg*n advocacy: An identity-based motivational account

**DOI:** 10.3389/fpsyg.2022.996250

**Published:** 2022-11-30

**Authors:** Ben De Groeve, Brent Bleys, Liselot Hudders

**Affiliations:** ^1^Center for Persuasive Communication, Department of Communication Sciences, Ghent University, Ghent, Belgium; ^2^Department of Economics, Ghent University, Ghent, Belgium; ^3^Department of Marketing, Innovation and Organisation, Ghent University, Ghent, Belgium

**Keywords:** animal-product consumption, identity and conflict, motivated cognitions, carnism, dietary change, veg*nism

## Abstract

Animal-based diets in Western countries are increasingly regarded as unsustainable because of their impact on human health, environmental and animal welfare. Promoting shifts toward more plant-based diets seems an effective way to avoid these harms in practice. Nevertheless, claims against the consumption of animal products contradict the ideology of the omnivorous majority known as carnism. Carnism supports animal-product consumption as a cherished social habit that is harmless and unavoidable and invalidates minorities with plant-based diets: vegetarians and vegans (veg*ns). In this theoretical review, we integrate socio-psychological and empirical literature to provide an identity-based motivational account of ideological resistance to veg*n advocacy. Advocates who argue against the consumption of animal products often make claims that it is harmful, and avoidable by making dietary changes toward veg*n diets. In response, omnivores are likely to experience a simultaneous threat to their moral identity and their identity as consumer of animal products, which may arouse motivations to rationalize animal-product consumption and to obscure harms. If omnivores engage in such motivated reasoning and motivated ignorance, this may also inform negative stereotyping and stigmatization of veg*n advocates. These “pro-carnist” and “counter-veg*n” defenses can be linked with various personal and social motivations to eat animal products (e.g., meat attachment, gender, speciesism) and reinforce commitment to and ambivalence about eating animal products. This does not mean, however, that veg*n advocates cannot exert any influence. An apparent resistance may mask indirect and private acceptance of advocates’ claims, priming commitment to change behavior toward veg*n diets often at a later point in time. Based on our theoretical account, we provide directions for future research.

## Introduction

In Western countries, animal-based diets – i.e., diets centered around meat and other animal products (e.g., seafood, dairy, eggs) – are the norm and these diets are now spreading across the globe. This trend, however, is increasingly criticized by scientists (Graça, 2016; Ripple et al., 2017; Poore and Nemecek, 2018; Willett et al., 2019) and minorities with plant-based diets – i.e., diets centered around food derived from plants (e.g., vegetables, fruits, legumes, seeds, nuts) (Melina et al., 2016; Rosenfeld, 2018; Medawar et al., 2019). Although “plant-based diets” is an umbrella term that may include diets with fewer animal products (Hemler and Hu, 2019), the most prominent and norm-challenging plant-based diets are those of vegetarians (who eschew meat) and vegans (who eschew animal products in general; Rosenfeld, 2018).

Vegetarians and vegans (veg*ns) often oppose the consumption of animal products because of their impact on animal welfare, environmental sustainability and human health (Janssen et al., 2016; Rosenfeld, 2018; Hopwood et al., 2020). In the following sections, we discuss these three common “veg*n” motives as claims against animal-product consumption (§1.1). Next, we discuss resistance to veg*n dietary change among the omnivorous majority, including identity-based motivational resistance (§1.2). We then clarify the aim and structure of our article (§1.3).

### Claims against animal-product consumption

#### Animal products and animal welfare

Because the animals farmed for food (chickens, pigs, ruminants, fish) are most probably sentient and able to suffer (Low, 2012; Fleischman, 2020), their mass production and instrumental use for consumption poses a pressing moral problem (Singer, 1975; Francione, 2020; Bruers, 2021). At any given moment, there are billions of vertebrate animals that are being farmed for food globally and most are raised in factory farms to maximize productivity (~74% of farmed land animals and virtually all farmed fish; Anthis & Anthis, 2019). Common sources of animal suffering include: intensive confinement in artificial conditions, unhygienic overcrowding, early mother-offspring separation and mutilating procedures (e.g., debeaking of chickens, tail docking of pigs, disbudding of cattle; Graça, 2016; Nordquist et al., 2017). Even “humane” slaughter typically involves stunning by a captive bolt, through electrocution or gas suffocation (Browning and Veit, 2020). To deny sentient beings bodily autonomy and care simply because they do not belong to the human species would be arbitrary species-based discrimination (i.e., speciesism; Bruers, 2021). Boycotting products for which animals were exploited and harmed by adopting a vegan lifestyle “as far as possible and practicable” seems to be a logically consistent approach to avoid speciesism and prevent suffering (Wrenn, 2018; Francione, 2020; Bruers, 2021). Likewise, avoiding the killing (i.e., vegetarianism) and exploitation of farmed animals (i.e., veganism) for consuming their flesh and byproducts as food is often a primary motive of veg*ns (Janssen et al., 2016; Rothgerber, 2017). Although animal rights arguments arguably provide the clearest challenge against animal-product consumption, the (over)consumption of animal products also poses environmental and health problems (Clark et al., 2019; Willett et al., 2019).

#### Animal products and environmental sustainability

Indeed, the widespread global consumption of animal products, particularly in Western countries, is a leading cause of urgent environmental problems, including the decimation of natural habitats and wildlife, nutrient pollution and global warming (Machovina et al., 2015; Springmann et al., 2016; Poore and Nemecek, 2018). While environmental impacts may vary considerably depending on the type of animal product and the producer (up to 50-fold for the same product; Poore and Nemecek, 2018), plant-based foods are generally less resource-intensive (excl. nuts, legumes) and polluting (Poore and Nemecek, 2018; Shepon et al., 2018), especially compared to red and ruminant meats (10–100 fold impact; Clark et al., 2019, 2022). Likewise, diets with less animal products (e.g., healthy meat-reduced, no ruminant meat, veg*n) offer substantial environmental benefits, with vegan diets being the most eco-friendly (Hallström et al., 2015; Chai et al., 2019). Although modern plant-based diets increasingly include highly processed animal-product alternatives (e.g., sausages, burgers), which are usually more impactful than minimally processed plant foods (MacDiarmid, 2021), actual animal products are overall still less environmentally sustainable than these alternatives (Bryant, 2022; Clark et al., 2022). Only in very rare cases a healthy diet with some meat (mainly local) is more sustainable than a veg*n diet (e.g., many processed foods from afar) (Chai et al., 2019). Directly allocating more plant crops for human consumption rather than feeding livestock would allow to reduce global food-feed competition and foster intra- and intergenerational equity while maintaining land to conserve biodiversity and regaining land to tackle climate change (Stoll-Kleemann and Schmidt, 2017; Poore and Nemecek, 2018).

#### Animal products and human health

Lastly, an excessive consumption of animal products that include high levels of saturated fat and cholesterol has been associated with chronic non-communicable diseases of welfare that lower life expectancies (Springmann et al., 2016; Wang et al., 2016; Clark et al., 2019; Barnard and Leroy, 2020). In particular, higher intakes of (un)processed red meat have been linked with cardiovascular disease (Wang et al., 2016), stroke (Kim et al., 2017), cancer (Wang et al., 2016), obesity (Rouhani et al., 2014) and type 2 diabetes (Neuenschwander et al., 2019). Nevertheless, the exact health effects of high meat consumption are difficult to disentangle because of potential confounding with (other) unhealthy behaviors (Boutron-Ruault et al., 2017). By contrast, fish and seafood are typically associated with improved health (Clark et al., 2019, 2022), though the (over)exploitation of wild-caught sea-animals and aquaculture expansion also poses environmental and animal welfare problems (Lam, 2019). In addition, avoiding factory farmed animal products (esp. from chickens and pigs) may decrease the risk of spreading zoonotic infectious diseases (Karesh et al., 2012; UNEP, 2020; Hayek, 2022) and antibiotic-resistance related illness (Tang et al., 2017; Hayek, 2022).

A common motive among people to adopt veg*n diets is to prevent and treat diseases of welfare (e.g., obesity, type 2 diabetes, cardiovascular disease) (Radnitz et al., 2015; Cramer et al., 2017; Costa et al., 2019) and nutritional science indicates that well-planned veg*n diets may indeed serve this function (Melina et al., 2016; Medawar et al., 2019; Selinger et al., 2022). Nevertheless, it is important to note that plant-based diets can also be unhealthy if they include unhealthy ingredients (e.g., highly processed plant-based alternatives high in fat, sugar and salt) or exclude healthy plant-based foods (e.g., vegetables, fruits, whole grains, olive oil, nuts) (Clark et al., 2019; Barnard and Leroy, 2020; MacDiarmid, 2021). There is also weak evidence that a vegan diet increases the risk of bone fractures, which could be due to lower intakes of vitamin B-12, vitamin D, calcium and protein (Craig, 2009; Selinger et al., 2022). Vegan diets thus require a reliable source of these nutrients *via* fortified foods or supplements (other nutrients of potential concern are omega-3-fatty acids, taurine, iron and zinc) (McCarty, 2004; Craig, 2009). Another common health motive among veg*ns is the promotion of physical and mental fitness (e.g., lose weight and gain energy) (Radnitz et al., 2015; Cramer et al., 2017; Costa et al., 2019). Evidence as to whether veg*n diets improve or decrease mental health (e.g., depression) is, however, mixed and not robust (Rosenfeld, 2018; Selinger et al., 2022).

Although veg*n diets could in principle provide integrated solutions to avoid animal, environmental and health harms associated with animal-product consumption, veg*ns remain a minority and claims against animal-based diets are often resisted by members of the omnivorous majority (Morris et al., 2014; De Groeve and Rosenfeld, 2022). This resistance has sparked a lot of academic interest (Graça et al., 2019).

### Resistance to veg*n dietary change

#### General barriers to veg*n dietary change

Over the past decades, a vast body of literature has emerged on the barriers and enablers to eat less animal products and to adopt veg*n diets (Corrin and Papadopoulos, 2017; Graça et al., 2019; Taufik et al., 2019), with important work on the history of meat-eating (Leroy and Praet, 2015; Chiles and Fitzgerald, 2018), reviews on the psychology of veg*nism (Ruby, 2012; Rosenfeld, 2018) and systematic reviews on interventions to reduce meat eating (Bianchi, Dorsel, et al., 2018a; Bianchi, Garnett, et al., 2018b; Harguess et al., 2020; Kwasny et al., 2022). Other reviews discussed meat reduction or plant-based diets/alternatives within the context of health promotion (Corrin and Papadopoulos, 2017; Bryant, 2022), pro-environmentalism (Hartmann and Siegrist, 2017; Bryant, 2022) and animal protection (Mathur et al., 2021). In addition, various theories have been applied to examine meat-eating (Povey et al., 2001; Graça et al., 2016; Grünhage and Reuter, 2021), including cognitive dissonance theory (Festinger, 1962) to gain insight in the meat paradox (“how can people care about animals, but also eat them?”) (Loughnan et al., 2014; Lin-Schilstra and Fischer, 2020) and the psychology of meat-eating as a morally questionable and dissonance-arousing activity (Bastian and Loughnan, 2017; Rothgerber, 2020). This vast literature indicates that the promotion of meat reduction and veg*n dietary changes is held back by a complex and diverse set of barriers, involving both macro-level historical, economic, political, technological and societal barriers and micro-level psychological barriers concerning awareness and habitual behavior, conflicting goals and values, ambivalent feelings and moral disengagement (Graça, 2016; Graça et al., 2019; Harguess et al., 2020).

More in particular, vegetarian and especially vegan diets may be criticized for being a privilege that may not be achievable for everyone due to medical conditions (e.g., health disorders), increased vulnerability (e.g., childhood, pregnancy), restricted food access (e.g., food deserts, livestock dependency), a lack of nutrition literacy, time or money (Greenebaum, 2017; Leroy et al., 2020). Cooking with fresh plant foods may be time-consuming while highly processed plant-based convenience foods are less nutritious in comparison (MacDiarmid, 2021). In addition, besides veg*n dietary changes, “less but better” animal products (i.e., more healthy, eco- and animal-friendly) and production systems (e.g., agroecological, regenerative) could provide pragmatic solutions to improve the global food system and human, environmental and animal health (Sahlin and Trewern, 2022).

In response to these criticisms, one may argue that an insistence on 100% purity in vegan practice is counter-productive (*cf.* “as far as possible”) (Leenaert, 2017) and that the ability to make healthy food choices in general is a luxury that requires nutrition literacy (Greenebaum, 2017). In addition, Bryant’s (2022) meta-analysis indicates that plant-based convenience foods are generally more nutritious than the animal products they replace. However, nutritional profiles of plant-based alternatives are highly variable (see also Clark et al., 2022) and further improving their healthiness (e.g., reducing salt and increasing protein, iron, vitamin B-12 content), familiarity, price and sustainability is recommended. Lastly, the promotion of veg*n diets may be complemented with “less but better” strategies, although these strategies have been criticized for lacking clear implementation goals (Sahlin and Trewern, 2022), while veg*n diets provide clear goals that also challenge speciesism more strongly (Singer, 1975; Rosenfeld, 2019b). In either case, the extent with which animal-product consumption is avoidable in practice remains open for empirical research. Although it is clear that omnivores may resist dietary change due to a wide variety of factors, our article elaborates on one potent motivational barrier: identity.

#### Identity-based motivated resistance to veg*n dietary change

More recently, there has been an increased attention on the influence of *social identity* on attitudes toward meat reduction and veg*nism, for example by considering the influence of political identity (Dhont and Hodson, 2014), gender (De Backer et al., 2020), species (Leite et al., 2019) and cultural identity (Ruby et al., 2016). Social identity refers to one’s self-perception based on feelings of belonging to a particular social group (e.g., conservatives, females, humans) (Tajfel, 1972; Turner and Reynolds, 2010). According to social identity theory (Tajfel and Turner, 1979, 1986), people are able to flexibly construe themselves as individuals or group members across situations, depending on perceived similarities and differences in a social comparison context (Turner and Reynolds, 2010; Hogg, 2016). The theory postulates that people desire a positive and distinct identity which, through social comparison and identification, could explain why people are motivated to favor their own ingroup (i.e., ingroup favoritism) and discriminate against outgroups, even when they are categorized based on minimal criteria (e.g., preferring a painting of Klee vs. Kandinsky) (Abrams, 2001; Otten, 2016). Given that the mainstream ideology (called “carnism”) legitimizes the consumption of animal products (e.g., as normal, natural, and necessary) (Joy, 2009, 2018) and delegitimizes veg*n minorities and veg*n practices (Joy, 2009, 2018), the omnivorous majority may resist changing their diet because they are motivated to protect their identity as consumers of animal products when confronted with an “outgroup” of veg*n advocates. What is currently missing in literature, however, is a comprehensive account of how ideological resistance to veg*n advocacy can be traced back to identity-based motivations.

### Aim and structure of the present review

**Figure 1:**
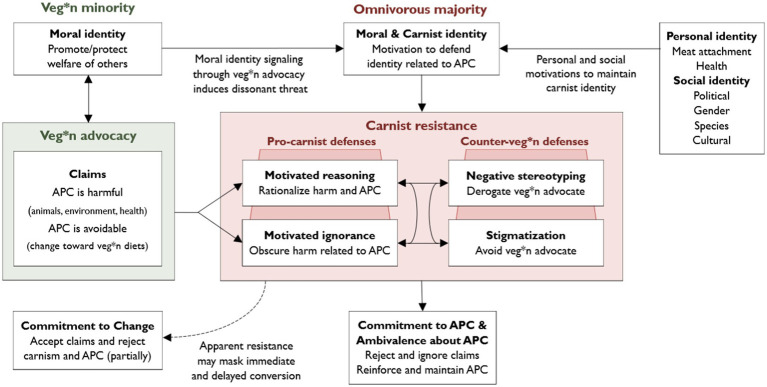
Identity-based Motivational Account of Carnist Resistance to Veg*n Advocacy. Veg*n minorities who engage in advocacy signal a moral identity by making claims that animal-product consumption (APC) is harmful and avoidable. This may evoke carnist resistance among the omnivirous majority, which may ultimately reinforce commitment to and ambivalence about APC. However, apparant resistance may mask immediate and delayed conversion (i.e., indirect and private acceptance of the advocates’ claims), priming commitment to behavioral change. We assume veg*n advocates’ primary claims consider the impact of APC on animal welfare, followed by concerns about environmental welfare and human health. Our account is explained in detail in the article.

The aim of this theoretical review is to provide an identity-based motivational account to understand ideological resistance to veg*n advocacy (visualized in Figure 1). First, we discuss veg*n advocacy against animal-product consumption (§2.1): Veg*ns often internalize their diet in their moral identity (§2.1.1), which may motivate them to engage in veg*n advocacy and signal a moral identity by making claims that eating animal products is harmful and avoidable (§2.1.2). Next, we introduce the reader to the omnivorous majority, which may exhibit ideological (i.e., carnist) resistance to veg*n advocacy (§2.2.1). We theorize that this “carnist resistance” stems from a simultaneous threat to omnivores’ moral identity and their identity as consumer of animal products (i.e., their carnist identity) (§2.2.2). To resolve moral/carnist identity threat, omnivores may rationalize their diet and obscure harms through motivated reasoning and motivated ignorance (i.e., pro-carnist defenses) (§2.2.3), which are theorized to inform negative stereotyping and stigmatization of veg*n advocates (i.e., counter-veg*n defenses), respectively (§2.2.4). We then discuss how these pro-carnist and counter-veg*n defenses are linked with different personal and social identity-based motivations (e.g., meat attachment, politics, gender) to maintain one’s carnist identity (§2.2.5) and how these defenses ultimately allow to reject and ignore advocate claims, reinforcing commitment to and ambivalence about animal-product consumption (§2.2.6). Nevertheless, this does not mean that advocates cannot exert influence. Apparent resistance to advocates’ claims may mask an indirect and private acceptance (i.e., conversion) and commitment to behavioral change (§2.3); this conversion can happen immediately (§2.3.1), but is often delayed (§2.3.2). Lastly, we provide directions for future research to test and qualify features of our account in the Discussion section (§3). One limitation we wish to disclose upfront, is that our work is mostly based on literature with WEIRD (i.e., Western, Educated, Industrialized, Rich, and Democratic) study samples and thus mainly representative for this study population.

## Theoretical account

### Veg*n advocacy against animal-product consumption

#### The veg*n minority and moral identity

As a minority (Martin et al., 2008), veg*ns are a numerically small group (about 2–10% in Western countries; Corrin & Papadopoulos, 2017) who typically hold moral, antinormative positions. Although veg*ns may have various motives for their diet – such as taste preference, religious or political beliefs, upbringing, influence of family and friends, and financial constraints (Ruby, 2012; Rosenfeld and Burrow, 2017), the three most common veg*n motives in Western countries include concerns about animal, environmental and health (see §1.1) (Janssen et al., 2016; Rosenfeld, 2018; Hopwood et al., 2020). These three motivations often co-occur (Janssen et al., 2016; Trethewey and Jackson, 2019) and may form a hierarchy with “more moral” motivations being viewed more positively (i.e., concerns about animals followed by environmental and then health concerns), especially among vegans (MacInnis and Hodson, 2021).

Because veg*ns often decide to consciously deviate from carnist norms based on strong moral motivations, they tend to strongly internalize their diet as an important aspect of who they are (Rosenfeld and Burrow, 2018; Rosenfeld, 2019b) and as a part of their *moral identity* (Chuck et al., 2016; Feinberg et al., 2019), i.e., their identity as a morally committed person and associated thoughts, feelings, and behaviors with regard to promoting or protecting the welfare of others (Aquino and Reed, 2002; De Groeve and Rosenfeld, 2022). The labels “vegetarian” and “vegan” may be a source of ingroup pride (Rosenfeld, 2018), facilitate cooperative group formation (Smaldino, 2019) and moral identity signaling (Aquino and Reed, 2002; Paxman, 2016), so that omnivores may readily perceive veg*ns as morally committed advocates who attract attention for “their” cause (Markowski and Roxburgh, 2019; De Groeve et al., 2021).

#### Veg*n advocacy and moral identity signaling

Indeed, there are a lot of actions veg*ns may partake to promote their diet and moralized identity, such as sharing messages on social media, signing petitions, donating money to campaigns and/or protesting (Thomas et al., 2019). Veg*ns may participate in various education and community engagement – from cooking and sharing veg*n food with others, to writing books or articles, to engaging in outreach (e.g., giving lectures, advertising stalls) (Chuck et al., 2016; Paxman, 2016). Yet, there is a lot of heterogeneity in how veg*ns construe their identity and engage in different forms of advocacy (Chuck et al., 2016; Paxman, 2016; Thomas et al., 2019). While only a radical minority engages in illegal actions such as clandestine investigations and animal rescue operations in pursuit of social change (Thomas et al., 2019), many veg*ns may detach from the veg*n label in some circumstances, see it as a personal burden, and are reluctant to discuss their dietary preferences with others (Chuck et al., 2016; Paxman, 2016; Rosenfeld and Tomiyama, 2019).

Based literature on veg*ns’ main motives and collective goals (MacInnis and Hodson, 2021; De Groeve and Rosenfeld, 2022), we presuppose that veg*n advocacy primarily involves claims that animal-product consumption harms animals, while claims about environmental and/or health harms are secondary. Stronger moral convictions, a stronger rejection of carnist and speciesist majority beliefs and a higher perceived inconsistency between moral vs. majority beliefs may motivate activism (Piazza et al., 2015; Harrington et al., 2022). In addition, Judge et al. (2022) showed that more principled convictions predict engagement in vegan advocacy *via* a stronger identification with other vegans and animals, perceived collective efficacy and moral outrage. Although pragmatic veg*n advocates may welcome incremental dietary changes (i.e., eating *less* meat/animal products rather than none) based on various motives, the desire to communicate a clear moral identity with consistent goals may cause veg*ns to dissociate from other self-identified veg*ns who do not share the same motives (e.g., categorizing health-motivated veg*ns as merely plant-based dieters) or calls for change (e.g., dismissing incremental changes as hypocritical) (Leenaert, 2017; MacInnis and Hodson, 2021; De Groeve and Rosenfeld, 2022). Advocates who clearly signal a moral identity are likely to evoke carnism-induced ideological resistance among members of the omnivorous majority (De Groeve and Rosenfeld, 2022; De Groeve et al., 2022).

### Carnist resistance to veg*n advocacy

#### The omnivorous majority and carnist resistance

Although humans have gradually included meat in their diet over evolutionary history, the prevalent consumption of animal products only became the norm in Western countries over the last century (Graça, 2016; Chiles and Fitzgerald, 2018). Joy (2009) introduced the term “carnism” to refer to the normative belief system that legitimizes animal-product consumption as a given rather than a choice, rendering associated harms “invisible.” Indeed, many people nowadays are socialized to adopt a diet rich in animal products as a part of their identity, which is by default deemed appropriate and therefore unlabeled (Bastian and Loughnan, 2017; de Boer et al., 2017). Consequently, omnivores generally do not consider their diet as a central aspect of who they are or take pride in their diet (Piazza et al., 2015; Rosenfeld and Burrow, 2018). Although omnivores generally care about animal welfare and, to some extent, the environment (Trethewey & Jackson, 2019), they tend to dissociate these values from their dietary pattern (Lacroix and Gifford, 2019; Rothgerber, 2020) and do not report prosocial/moral motives to follow their diet (Rosenfeld and Burrow, 2018). The most common reasons for eating meat include taste, habit, upbringing, convenience (e.g., socially, practically, financially), and perceived health of eating meat (Povey et al., 2001; Mullee et al., 2017; Lacroix and Gifford, 2019). Compared to veg*ns, omnivores are moderately more likely to endorse conventional values that bind groups together, including power/authority, loyalty and purity (Graham et al., 2013; Grünhage and Reuter, 2021; Holler et al., 2021).

Because the omnivorous diet is conventional, majority norms exert a strong immediate influence on omnivores. One reason for majority’s social power is that majority membership protects against social rejection (Martin et al., 2008): eating and sharing animal products is a way to facilitate social bonding and different animal foods may characterize different nations (e.g., Australian meat pies), celebrations (e.g., Thanksgiving Turkey) or (sub) cultures (Markowski and Roxburgh, 2019; Nguyen and Platow, 2021). For example, in Western countries many people love dogs and eat pigs (Joy, 2009), while eating dogs may be acceptable in some Asian countries (Podberscek, 2009) and eating pigs is forbidden by Islamic and Judaic scripture (Farouk et al., 2015). Majorities also exert a powerful influence because individuals may doubt their own convictions in the face of the majority (Martin et al., 2008; Bolderdijk and Jans, 2021; May and Kumar, 2022). Conforming to majority norms (e.g., eat what your peers eat) enables fast and frugal decision making, obviating the need for individuals to extensively deliberate on food choice (Henrich et al., 2001). Unsurprisingly then, one of the most persistent barriers to follow more plant-based diets are conformity pressures (Ruby, 2012; Leenaert, 2017; Lacroix and Gifford, 2019).

Conformity pressures may explain why meat reduction initiatives may evoke considerable resistance (Morris et al., 2014) and why omnivores often report having experienced conflict with veg*ns, who oppose the majority’s carnist ideology (Guerin, 2014; Piazza et al., 2015; Markowski and Roxburgh, 2019). Conversely, veg*ns can be targets of anti-veg*n bias (Earle and Hodson, 2017) such as social stigma and negative stereotypes (Chin et al., 2002; Minson and Monin, 2012; MacInnis and Hodson, 2017; Markowski and Roxburgh, 2019; De Groeve and Rosenfeld, 2022). Joy (2018) distinguishes two sets of psychological defenses people use to maintain animal-product consumption and resist change, which we refer to as “carnist resistance”: (1) defenses that legitimize the consumption of animal products (i.e., pro-carnist defenses) and (2) defenses that delegitimize veg*nism (i.e., counter-veg*n defenses). These two defenses resemble a tendency of people to selectively seek and process information that confirms one’s identity or position (i.e., confirmation bias), while being disproportionally more critical of refuting information (i.e., disconfirmation bias; Taber and Lodge, 2012). At the same time, it is important to note that defensive and stigmatizing attitudes are dynamic and may vary considerable across individuals, cultures and contexts (Paxman, 2016; MacInnis and Hodson, 2017). Individual variation and the versatility of the human mind (e.g., identities, language) through time should prevent us from essentializing identity categories. As we analyze carnist resistance further, we will consider more variety among omnivores in how they might respond to veg*n advocacy.

#### Carnist resistance as a consequence of moral-carnist identity threat

Most research that examined carnist resistance until now has relied on cognitive dissonance theory (Festinger, 1962) to explain the maintenance of meat consumption as a morally-conflicting behavior (i.e., the meat paradox) (Bastian and Loughnan, 2017; Lin-Schilstra and Fischer, 2020; Rothgerber, 2020). Cognitive dissonance refers to a state of negative arousal that arises when someone holds two contradictory cognitions, typically involving a behavior versus an attitude (e.g., eating meat but caring for animals). Subsequently, individuals are motivated to resolve this perceived inconsistency either by changing one’s behavior (e.g., refusing to eat meat) or by changing one’s attitudes (i.e., defending meat consumption).

In our account of carnist resistance, we draw on revisions of cognitive dissonance theory that integrate the role of identity or the self-concept (Cooper, 2007). Self-based revisions of dissonance theory assert that perceived attitude-behavior inconsistencies do not just arouse dissonance due to a perception of inconsistency between two cognitions (Festinger, 1962), but that dissonance only occurs to the extent that it involves a threat to the self (Cooper, 2007): When a behavior is perceived as contradicting one’s self-concept in Aronson’s (1968) self-consistency account, or when it challenges one’s self-integrity as a moral and competent person in Steele’s (1988) self-affirmation account. These accounts are in line with the postulate that humans desire a positive and distinct identity (Hogg, 2016). Consequently, omnivores are likely to experience self-threat when veg*n advocates signal a moral identity by claiming that animal-product consumption entails avoidable harm. More specifically, such an exposure may readily threaten omnivores’ own moral identity (Bastian and Loughnan, 2017; De Groeve and Rosenfeld, 2022). In addition, although omnivores generally view eating animal products as a given and not as central to their identity (Joy, 2009; Rosenfeld and Burrow, 2018), a confrontation with veg*n advocates may increase the salience of omnivores’ *carnist identity* – their identity as consumers of animal products (or non-veg*ns) and the thoughts, feelings and behaviors associated with it (De Groeve and Rosenfeld, 2022). Based on this theorizing, Minson and Monin (2012) their measure of anticipated moral reproach (e.g., “If they saw what I normally eat, most vegetarians would think I am extremely (im)moral.”) can be construed as a proxy of moral/carnist identity threat and the meat paradox can be construed as an inconsistency between omnivores’ moral and carnist identity, which omnivores are motivated to resolve in order to maintain a positive identity and avoid dissonant feelings of self-threat. Our theorizing is also consistent with the New Look model of dissonance, according to which individuals reduce dissonance to render consequences of behavior non-aversive (Cooper, 2007).

Based on research on the meat paradox, meat-related dissonance (e.g., Bastian and Loughnan, 2017; Rothgerber, 2020), moral disengagement (Graça et al., 2016) and research on identity-protective and motivated cognition (Kunda, 1990; Kahan, 2013; Williams, 2020; May and Kumar, 2022), a distinction can be made between two broad categories of pro-carnist defenses omnivores employ to resolve moral/carnist identity threat: (1) motivated reasoning and (2) motivated ignorance. Below, we will shortly discuss these motivated defenses and clarify their interrelationship with counter-veg*n defenses in the form of negative stereotyping and stigmatization. We note that our discussion is mainly focused on defenses against animal welfare claims because these embody the primary motive for veg*n advocacy (MacInnis and Hodson, 2021) and have been studied most extensively. In addition, environmental and especially health claims pro veg*nism may be less persuasive or arouse less dissonance (De Groeve and Rosenfeld, 2022; Silva Souza and O’Dwyer, 2022).

#### Pro-carnist defenses: Motivated reasoning and motivated ignorance

##### Motivated reasoning: Rationalize harm and animal-product consumption

When omnivores are exposed to veg*n advocates, we argue that they are likely to experience dissonance because their claims are difficult to ignore (De Groeve and Rosenfeld, 2022), subsequently arousing a motivation to actively defend their salient carnist identity by engaging in motivated reasoning (Rothgerber, 2020)*. Motivated reasoning* involves arriving at a particular position one wants to arrive at (Kunda, 1990), which allows for reducing dissonance (Rothgerber, 2020), affirming one’s identity (Kahan, 2013) and expressing loyalty to groups one depends on for material and social support (Kahan, 2013). Defense mechanisms relying on motivated reasoning justify eating animal-derived products as relatively harmless and/or as difficult or impossible to avoid, implying a denial of responsibility (Bastian and Loughnan, 2017). These dissonance-reducing defenses have also been described as unapologetic or direct/active defenses in literature on meat-related dissonance (Hartmann and Siegrist, 2020; Rothgerber, 2020) and distort evidence showing that eating animal products involves avoidable harm.

Omnivores may rationalize harm by denying the collateral damage associated with eating animals for the environment, public health and animal welfare (Rothgerber, 2013; Graça et al., 2016). Eating animal products may be rationalized as unavoidable (i.e., a requirement) by endorsing “the 4Ns” (Piazza et al., 2015, 2020; Hopwood et al., 2021a): the belief that consuming animal products is *Necessary* for one’s health, too *Nice* or enjoyable to forego, a *Normal* practice that is socially desirable and something *Natural* to do. Omnivores may also feel morally licensed to eat animal products if they endorse speciesism and human supremacy over animals and the natural environment (Graça et al., 2016; Caviola et al., 2019), for example through hierarchical and fate justifications (e.g., humans are on the top of the food chain and meant to eat animals) and religious licensing (i.e., God intended for us to eat animals; Rothgerber, 2013). Furthermore, omnivores may deny or diffuse responsibility by expressing moral outrage and blaming third parties such as industries, society and government (Graça et al., 2016; Rothgerber, 2020; Silva Souza and O’Dwyer, 2022).

These defensive rationalizations are reminiscent of a fight-response to stress (Cannon, 1932) and Joy’s (2009) characterization of carnism as a power-oriented ideology that supports a culture of violence. Various institutions are complicit by catering to omnivores’ confirmation bias: Animal farming industry and stakeholder groups have a powerful interest to externalize production costs and reinforce a cheap, ubiquitous supply of animal products (Weis, 2013); existing laws and advertisements often convey the falsely reassuring message that farmed animals are treated humanely without needless suffering (Bastian and Loughnan, 2017; Francione, 2020; Clare et al., 2022), and media coverage of veganism tends to confirm the ideological preferences of their audience (Cole and Morgan, 2011).

Although omnivores who are more committed to their diet are more likely to engage in motivated reasoning (Rothgerber, 2013, 2020; Piazza et al., 2015; Graça et al., 2016), it is relevant to note that rationalizations in support of animal-product consumption are typically not strongly endorsed by omnivores (Rothgerber, 2013; Piazza et al., 2015; Monteiro et al., 2017). This may indicate that these defenses mainly serve to maintain a mostly habitual activity once ambivalent thoughts or feelings about eating animal products come to mind (Buttlar and Walther, 2018; Piazza, 2020). Omnivores may also vary considerably in how they respond to veg*n advocates. In between radical vegan activists and deliberate anti-veg*ns at two opposing ends of a putative dietarian-ideological continuum (De Groeve and Rosenfeld, 2022; Gregson et al., 2022; Verain et al., 2022), omnivores’ attitudes may be less outspoken and more ambivalent (Povey et al., 2001; Berndsen and Van Der Pligt, 2004; Graça, Calheiros, et al., 2015a). For example, people who consciously eat less meat (i.e., flexitarians) may still belong to the omnivorous majority, but resemble veg*ns in that they deviate from carnist norms (Rosenfeld, 2018). Likewise, their attitudes toward meat and vegetarianism often fall in-between those of conventional omnivores and veg*ns (Rosenfeld, 2018) and flexitarians are less likely to defend meat-eating through motivated reasoning (Rosenfeld, 2018; Rosenfeld et al., 2019; De Groeve et al., 2022).

As motivated reasoning involves defending oneself using reasons irrespective of their accuracy (Williams, 2020) and arriving at a particular position one wants to arrive at (Kunda, 1990), it is typically related with a motivation to avoid acquiring certain information contradicting this position: motivated ignorance (Williams, 2020), most clearly expressed in the form of denial (Piazza et al., 2015; Rothgerber, 2020). However, if people are more ambivalent about eating animal products, motivated ignorance may suffice as a defense mechanism on itself without actively defending one’s carnist identity through motivated reasoning (Rothgerber, 2020).

##### Motivated ignorance: Obscuring harm related to animal-product consumption

Although motivated (or strategic) ignorance generally refers to an avoidance of acquiring available information that is perceived as potentially aversive, it may also involve the distortion or obfuscation of information (Onwezen and van der Weele, 2016; Golman et al., 2017), motivated forgetting, a refusal to acknowledge what one knows (willful ignorance), and self-deception (Golman et al., 2017). Identity-protective motivated ignorance may be socially adaptive, as it allows people to blend in with desirable groups and avoid social sanctions (Williams, 2020). Concerning animal-product consumption, defense mechanisms relying on motivated ignorance obscure evidence of harm related to animal-product consumption (Rothgerber, 2020). These defenses are also described as dissonance-preventing, indirect or apologetic defenses in literature on meat-related dissonance (Hartmann and Siegrist, 2020; Rothgerber, 2020), allowing omnivores to avoid carnist identity threat and comply with the omnivorous majority.

Motivated ignorance is evident in omnivores who avoid information about the sentient minds of farmed animals (Buttlar and Walther, 2018; Leach et al., 2022) and factory farming conditions (Cornish et al., 2016; Onwezen and van der Weele, 2016). Consumers may also dissociate vegan diets from animal rights philosophy (Lundahl, 2020) and animal products from their animal origins so that farmed animals and their suffering remain hidden (Benningstad and Kunst, 2020). Animal harms can also be obscured if consumers dichotomize animals in those who are farmed for food (i.e., treated as objects) and those who are kept as companion animals (i.e., treated as subjects) (Amiot et al., 2019; Rothgerber, 2020) or if harm is neutralized by claiming that meat is only rarely eaten or ethically sourced (Rothgerber, 2015, 2020; Dowsett et al., 2018). For example, a recent US survey found that, while consumers on average believed that 69% of animals are factory farmed, many reported thinking that animals are treated well (62%) and that they usually buy animal products from humanely raised animals (Reese, 2021). Evidence of a rising flexitarian self-identification combined with stable and high self-reported meat consumption levels has been reported for the Netherlands (Dagevos, 2021). Another recent study showed that consumers may willfully disregard solutions targeting factory farming to prevent future pandemics, especially if they are meat-committed (Dhont et al., 2021). Socially motivated ignorance and fear of ostracism could play a role in climate change skepticism in (conservative) groups where expressing concern about global warming is identity-inconsistent (Williams, 2020). Socially motivated ignorance may reinforce pluralistic ignorance, a situation where individuals privately reject a norm, but are swayed to comply with the majority position because they falsely assume that others privately endorse it (Delon, 2018). In this way, omnivores can (privately) identify as being animal-and eco-friendly (Trethewey and Jackson, 2019) or morally condemn conventional farming conditions when reading about it (Hartmann and Siegrist, 2020) without considering themselves responsible for its problems (Graça et al., 2016). A considerable amount of US consumers even favors banning factory farming (51%), slaughterhouses (45%) or all animal farming (36%) (Reese, 2021), while not adopting congruent dietary behavior that may reduce ambivalent feelings about eating meat (Povey et al., 2001).

Defenses relying on motivated ignorance are reminiscent of a flight-response to stress (Cannon, 1932) and Joy’s (2009) characterization of carnism as an “invisible” ideology that supports a culture of silence where the implicit norm is to speak no harm, hear no harm and see no harm. How people produce, promote, prepare and talk about animal products obscures the link between the product and its animal origins (Benningstad and Kunst, 2020). For example, meat consumers may feel more apathy toward animals and feel less disgusted by eating meat if the killing of farmed animals is described as “harvesting,” if the flesh of animals (pigs, cows) is described in culinary terms (pork, beef), or if the meat resembles the original animal less rather than more (Kunst and Hohle, 2016). Animal farming industry uses similar tactics as the tobacco and fossil industry to mystify harm, while encouraging ongoing consumption (Clare et al., 2022). Bastian and Loughnan (2017) elaborate on how information avoidance may spread and become embedded in minds and cultures and how habits, institutions and rituals may operate like a veil of ignorance. In what follows, we discuss how motivated cognitions (i.e., pro-carnist defenses) among omnivores may reinforce the negative stereotyping and stigmatization (i.e., counter-veg*n defenses) of veg*n advocates who pierce this veil of ignorance by challenging animal-product consumption (Rothgerber, 2020; De Groeve and Rosenfeld, 2022).

#### Counter-veg*n defenses: Negative stereotyping and stigmatization of veg*n advocates

##### Motivated reasoning informs negative stereotyping of veg*n advocates

According to self-categorization theory (Turner et al., 1987; Hogg, 2016), stereotypes are not just mental representations of a social category (i.e., prototypes) that are widely shared among people, but also serve the social function to justify ingroup behavior (Hornsey, 2008; Turner and Reynolds, 2010). As such, negative stereotypes that derogate the veg*n outgroup can be connected with motivated reasoning to justify one’s carnist identity and diet. Although the content of stereotypes typically revolves around a stable core (e.g., vegetarians do not eat meat), their expression may differ depending on the social comparison context (Hogg, 2016). For example, (negative) stereotyping may depend on how visible or voluntary one’s veg*n identity is or on the extent that a veg*n identity is seen as socially disruptive or threatening (Greenebaum, 2012; Minson and Monin, 2012; Guerin, 2014; Rothgerber, 2014).

Although veg*ns may be appreciated for their perceived morality, commitment and their animal-loving, eco-friendly and healthy image (De Groeve et al., 2021), arguably the most salient negative stereotype associated with veg*n identities, is that they are moralistic (Markowski and Roxburgh, 2019; De Groeve et al., 2021; De Groeve and Rosenfeld, 2022). This moralistic stereotype reflects a social truth to some extent, because veg*ns may generally look down on omnivores more than omnivores look down on veg*ns (Rosenfeld and Burrow, 2018; Rosenfeld, 2019a), arguably because they are more likely to strongly identify as a group challenging (vs. defending) the status quo (Bäck and Lindholm, 2014) and view the consumption of animals for food as immoral and disgusting (Povey et al., 2001). Similarly, vegans may negatively judge vegetarians as hypocrites and akin to omnivores for still consuming dairy and eggs (thus supporting the exploitation of cows and chickens) (Povey et al., 2001; Ruby, 2012; Plante et al., 2019). Nonetheless, research also suggests that omnivores may overestimate the extent with which vegetarians look down on them and a stronger anticipated moral reproach predicts more negative stereotyping (Minson and Monin, 2012). Omnivores’ moralistic perceptions of veg*ns may partly stem from defensively distorting moral commitment perceptions to resolve the meat paradox and carnist identity threat (De Groeve and Rosenfeld, 2022). Omnivores are more likely to stereotype vegans (vs. vegetarians) as moralistic (De Groeve et al., 2021), especially if vegans have animal ethics (vs. health) motivations and engage in public advocacy (De Groeve et al., 2022), and if veg*ns’ communication is static and results-oriented rather than dynamic and process-oriented (Weiper and Vonk, 2021).

Although moralistic stereotypes appear to be the most pervasive, De Groeve and Rosenfeld (2022) argue that the rationalization that animal-product consumption is relatively harmless supports the stereotyping of veg*ns as overly sensitive and effeminate, while 4Ns endorsements that make animal-product consumption seem practically unavoidable may be reinforced by stereotyping veg*ns as opposing the Ns: weird, eccentric and unsociable (not normal), too boring (not nice), unnatural (not natural), hypocritical and unhealthy (by opposing the claimed nutritional necessity of animal products). Just like motivated reasoning can be seen as a manifestation of motivated ignorance, negative stereotyping is but one expression of stigmatization, and studies reveal that veg*n stigma and motivated ignorance about the harms related to animal products are interconnected (Markowski and Roxburgh, 2019; Rothgerber, 2020).

##### Motivated ignorance informs stigmatization of veg*n advocates

To stigmatize someone, is to identify them as deviant, label them and negatively stereotype them, which serves to otherize and discriminate individuals as outgroup members, resulting in a “spoiled” identity and status loss for the stigmatized (Link and Phelan, 2001; Major and O’Brien, 2005). Put differently, stigmatized individuals are socially marked as unaccepted and to be avoided (Kurzban and Leary, 2001). Arguably the most extensive study examining stigmatization of veg*ns was conducted by MacInnis and Hodson (2017), who showed that veg*ns, in particular vegans, were rated equivalently or significantly more negatively than other targets of prejudice (e.g., Black people). People were more likely to avoid veg*ns in general, as friends or as potential partners if they more strongly identified as meat-eaters. Conversely, 46% of vegetarians and 67% of vegans reported some level of discrimination in their lives and some vegans even reported decreased contact with friends (25%) and family (10%) after disclosing being vegan. Veg*ns often engage in stigma/impression management strategies to navigate and smoothen social interactions with omnivores and present their identity in a more positive light (Greenebaum, 2012; Paxman, 2016; Rosenfeld and Tomiyama, 2019), for example by selectively disclosing their identity and communicating strategically about their diet to avoid defensiveness or feelings of guilt among omnivores. Despite clear evidence of stigmatization, we reiterate that this is a dynamic context-dependent phenomenon. In general, views of veg*ns are often rather positive, yet mixed and more negative toward vegans (Corrin and Papadopoulos, 2017; De Groeve et al., 2021), resembling ambivalent feelings toward meat (van der Weele, 2013; Graça, Oliveira, et al., 2015b).

In the context of veg*n advocacy, stigmatization allows omnivores to resist and avoid advocate claims to maintain their carnist identity (Markowski and Roxburgh, 2019). Zane et al. (2016) directly demonstrated a link between stigmatization and motivated ignorance by showing that consumers who willfully ignored ethical product information derogated consumers who did inform themselves before purchasing products. Likewise, the derogation of veg*ns (Minson and Monin, 2012) can be traced back to motivated ignorance among omnivores about the moral implications of their diet (Rothgerber, 2020). By “shooting the (veg*n) messenger” (Joy, 2018) or “condemning the condemner” (Cole and Morgan, 2011; Rothgerber, 2020) omnivores may deflect attention from messages that morally condemn their dietary behavior and carnist identity (De Groeve and Rosenfeld, 2022). Likewise, Cole and Morgan (2011) interpreted evidence of vegan stigma in UK national newspapers as a reflection of motivated ignorance about the ethics of exploiting and killing animals. The link between stigmatization and motivated ignorance is also vividly expressed by an omnivorous participant in Guerin’s (2014) focus group study: *“I do not want people to get in my face and tell me the gory details of where meat comes from while I’m eating a burger. I mean, I’ve never been pressured to stop eating it or anything but I would probably just be put-off and ignore them.”* (p. 16). By voicing concerns about people pushing against meat, omnivores may mark vegetarian advocates as ignorable. Conversely, focus group studies among veg*ns also provide vivid examples of the link between stigmatization and motivated ignorance (Greenebaum, 2012), as one vegetarian notes: *“I learned along the way that the majority of people have no idea how the animal gets to that plate. They are just completely ignorant about that. And when I start talking about it they just tell me to shut up.”* (p. 315). Although actively derogating veg*ns by voicing negative stereotypes provide the clearest example of stigmatization, it can also be expressed as passive avoidance (e.g., decreased contact family and friends) (MacInnis and Hodson, 2017). Ultimately, we theorize that the stigmatization and negative stereotyping of veg*ns discussed above serve to protect personal and/or social motivations tied with one’s carnist identity. Below, we discuss how pro-carnist and counter-veg*n defenses can be linked with some of the most potent personal and social identity-based motivations to maintain a carnist identity.

#### Pro-carnist and counter-veg*n defenses: Personal and social identity-based motivations

##### Personal identity

###### Meat attachment

Veg*n advocates may pose a threat to the self-interest in maintaining a carnist identity. Self-interest, which is often connoted with hedonistic attachment, forms an obvious barrier against moralizing animal-product consumption and making personal sacrifices for the common good (Feinberg et al., 2019). People who eat more meat and identify more strongly as a meat-eater tend to have a stronger personal attachment to eating meat, causing them to morally disengage from meat production harms (Graça et al., 2016) through motivated reasoning to justify meat (Piazza et al., 2015; Verain et al., 2022) and motivated ignorance of animal minds (Leach et al., 2022), dismissive reactions toward meat substitution (Graça et al., 2016) and stigmatizing attitudes toward veg*ns (Dhont and Hodson, 2014; Earle and Hodson, 2017; Vandermoere et al., 2019). Those who are less attached to meat and more willing to change their diet (e.g., flexitarians) are less likely to engage in motivated reasoning and negative stereotyping of veg*ns (Minson and Monin, 2012; De Groeve et al., 2022).

###### Health

Healthy eating may also be a personal motive to eat meat. Nevertheless, previous studies found that identifying oneself as a healthy eater does not predict self-reported meat consumption (Trethewey and Jackson, 2019) or intentions to eat (less) red meat (Carfora et al., 2017), but that it does predict fruit and vegetable intake (Carfora et al., 2016) and intentions to follow vegetarian or plant-based diets (Povey et al., 2001; Graça et al., 2019). Although veg*ns and plant-based foods are often perceived as healthy, veg*nism may be perceived as unhealthy to the extent that animal products are seen as more nutritionally adequate or necessary (De Groeve and Rosenfeld, 2022; Gregson et al., 2022). In addition, more processed foods are generally seen as less healthy, which poses a barrier for promoting (healthy) plant-based animal-product alternatives (Bryant, 2022; Hartmann et al., 2022).

##### Social identity

###### Political identity

Conservatives may be more socially motivated to maintain their carnist identity. People who identify as meat-eaters more strongly and eat more meat are more likely to endorse conservativism (Allen and Ng, 2003; Dhont and Hodson, 2014), typically characterized as two dispositional tendencies: (a) right-wing authoritarianism (RWA, i.e., a preference for tradition and punishment of non-conformists), which predicts a higher endorsement of conventional values (i.e., authority, loyalty, purity), and (b) social dominance orientation (SDO, i.e., a preference for hierarchical domination over lower-status groups), which predicts a lower endorsement of a postconventional, universal morality that prioritizes the welfare of individuals (i.e., harm avoidance, justice) (Federico et al., 2013; Dhont and Hodson, 2014; Kugler et al., 2014). Conservatives may partly identify as meat-eaters more strongly because veg*ns pose a threat to traditional ways of life *via* RWA (Dhont and Hodson, 2014; Judge and Wilson, 2019; Leite et al., 2019) and because meat – in particular red meat – symbolizes power, inequality, and human supremacy over nature and animals *via* SDO (Allen and Ng, 2003; Dhont and Hodson, 2014). Veg*ns’ status as egalitarian norm-violators – reflected by stereotypes that they are liberal, hippies and pacifists (Sadalla and Burroughs, 1981; Minson and Monin, 2012; De Groeve et al., 2021) – may generate pushback against them to defend the dominant carnist ideology (Dhont and Hodson, 2014; MacInnis and Hodson, 2017; Monteiro et al., 2017). Given that conservatives often use moralistic stereotypes (e.g., social justice warrior, snowflake) as slurs against progressive ideas (Prażmo, 2019), conservatives may be more likely to view veg*ns as arrogant competitors overcommitted to change society (Dhont and Hodson, 2014; Judge and Wilson, 2019). Research among current and former vegetarians shows that those higher on conservativism are significantly more likely to have lapsed into meat-eating, mainly because of lower social justice motivations, but also because of a lack of social support (Hodson and Earle, 2018). Given that conservativism has also been analyzed as a motivated social cognition that varies situationally (not only dispositionally) to deal with uncertain, dangerous (*cf.* RWA) and competitive (*cf.* SDO) environments (Jost et al., 2003; Sibley and Duckitt, 2013) and that animal-product consumption remains widespread, liberals may resemble conservatives in their resistance to advocacy and dietary change. Nevertheless, liberals generally feel less threatened by veg*ns (MacInnis and Hodson, 2017) and liberals and centrists who eat more meat may be more likely to exhibit motivated ignorance (avoidance, dissociation and dichotomization; Grünhage & Reuter, 2021). A lower meat consumption and veg*nism has been associated with a higher endorsement of universal values, empathy and openness (Keller and Siegrist, 2015; Holler et al., 2021), which oppose SDO and RWA (Sibley and Duckitt, 2013).

###### Gender identity

Veg*n advocates may also pose a threat to masculine identities. Across cultures, eating meat – in particular red meat – is linked with traditional notions of masculinity, which assert that “real” men are strong, virile and emotionally stoic (Rothgerber, 2013). Consequently, men may be socially motivated to show off their meat consumption to signal their masculinity in particular situations (Rothgerber, 2013; Rosenfeld, 2018). Omnivores, in particular omnivorous men, may rate vegetarian men more negatively than vegetarian women, arguably because vegetarianism is incongruent with traditional masculinity (MacInnis and Hodson, 2017; Rosenfeld, 2018). For instance, the link between meat and “masculine” values to be dominant and physically strong contrasts with the lower social status of veg*n minorities and their reputation as being physically weak and sentimental (Rothgerber, 2013; Corrin and Papadopoulos, 2017). Men, especially those who endorse traditional masculinity, rationalize meat-eating more (Hinrichs et al., 2022) and may derogate vegetarians to avoid appearing emasculated or feminine (Ruby and Heine, 2011; MacInnis and Hodson, 2017), though promoting plant-based eating does not necessarily increase defensiveness (Hinrichs et al., 2022). Traditional masculinity can be juxtaposed with new forms of masculinity characterized by valuing authenticity, holistic self-awareness and nurturing, and questioning male norms and privileges (De Backer et al., 2020). A stronger endorsement of new masculine values predicts a lower meat attachment and more positive attitudes toward vegetarians (De Backer et al., 2020). Compared to men, women are generally more willing to reduce their meat intake or be(come) veg*n (Ruby, 2012; Rosenfeld, 2018). Overall, women (vs. men) are more health-conscious (VanHeuvelen and VanHeuvelen, 2019), endorse universal values more strongly (Hayley et al., 2015; Rosenfeld, 2018) and are more likely to report that eating meat is unhealthy and harms the environment and animals (Mullee et al., 2017; Possidónio et al., 2019). Women are less likely to defend their diet through motivated reasoning (dissociation and avoidance are more common) (Rothgerber, 2013, 2020) and to stigmatize vegetarians (Vandermoere et al., 2019) and more likely to admire vegetarians (Ruby et al., 2016). Some studies, however, only found small or negligible gender differences in stereotyping (De Groeve et al., 2021, 2022).

###### Species identity

Veg*n advocates may also evoke resistance because their diets challenge speciesism and human supremacy, i.e., the belief that humans are distinct from and superior to non-human animals (Dhont & Hodson, 2014; Leite et al., 2019; Caviola et al., 2019, 2022). Meat-attached people are more likely to endorse human supremacy (Graça, Calheiros, et al., 2015a), which predicts a willingness to exploit animals and eat more meat (Dhont and Hodson, 2014). Monteiro et al.’s (2017) carnism measure, which seems to combine human supremacy beliefs (“carnistic dominance”) and meat-eating justifications (“carnistic defense”), is strongly correlated with seeing vegetarianism as a cultural threat, suggesting that omnivores who strongly endorse human supremacy and speciesism are more likely to defend animal-product consumption through motivated reasoning (see also Piazza et al., 2015; Graça et al., 2016). While vegetarianism threat increases negative feelings about eating meat, human supremacy and 4 N endorsement may alleviate such feelings (Amiot et al., 2019). Prejudiced attitudes toward animals and veg*ns can be explained by SDO (Dhont and Hodson, 2014), which is a common denominator of prejudices toward human outgroups (e.g., sexism, racism and other dehumanizing tendencies) (see the SD-HARM model; Dhont et al., 2016). In contrast, people who identify more strongly with animals are more likely to reject speciesism and justifications of animal use (Amiot and Bastian, 2017). Liberals (vs. conservatives), women (vs. men) and those who have more contact or affinity with animals (through pet ownership) are more likely to express positive affiliation with animals (Amiot and Bastian, 2017; Possidónio et al., 2019; Amiot et al., 2020; Rothgerber, 2020), which may have downstream positive effects on attitudes toward veg*ns (Earle et al., 2019; Leite et al., 2019; Hodson et al., 2020) with the caveat that derogating veg*ns would be more likely if omnivorous animal lovers feel that their moral self-concept is on the line (Minson and Monin, 2012).

###### Cultural identity

Lastly, eating animal foods may be an important part of one’s cultural identity. Nevertheless, psychological research on the role of culture in shaping one’s dietary identity, and attitudes toward animal products and veg*ns is scarce (Rosenfeld, 2018; Rothgerber, 2020). One study has shown, for example, that a higher national identification among Americans, Brits, and Australians predicted higher intentions to eat meat, and lower intentions to eat a vegetarian meal when eating meat is considered typical for one’s nation (Nguyen and Platow, 2021). In addition, attitudes in favor of beef have been shown to systematically predict anti-vegetarian prejudice among college students in Argentina, Brazil, France and the US (Earle and Hodson, 2017), with varying attitudes between these countries (Ruby et al., 2016). Concerning the role of religion, Rothgerber (2013) found that meat consumption frequency modestly correlates with religious justifications (e.g., God intended for us to eat animals), which are in turn associated with hierarchical and fate justifications, the endorsement of masculine norms among men and denying animal suffering (Rothgerber, 2013). This suggests, in line with vegetarian ecofeminist theory (Gaard, 2002), that patriarchal dualist religions may tie conservative, masculine, and human supremacist identities together in opposing veg*n advocacy. On the other hand, though, religious viewpoints are likely to be diverse; religious people may also view veg*nism as a sign of devotion and spiritual purity (Wrenn, 2019), in line with the Garden-of-Eden ideal (Bekoff and Meaney, 1998). Notably, in India, vegetarianism is part of religious traditions (i.e., Jainism, Hinduism) and vegetarians are *more* likely to endorse conservative values than omnivores (Ruby et al., 2013).

In sum, despite the existence of favorable attitudes toward veg*ns, omnivores who are confronted with veg*ns may become personally and/or socially motivated to defend their salient carnist identity by engaging in pro-carnist and counter-veg*n defenses. These defenses reinforce commitment to and ambivalence about animal-product consumption as a result.

#### Commitment to and ambivalence about animal-product consumption

Omnivores who are already committed to their diet and have a stronger carnist identity (typically more meat-attached, conservative, traditional men, speciesist and/or proud of their cultural identity) are more likely to actively defend themselves through motivated reasoning and negative stereotyping of veg*ns, which reinforces the idea that eating (more) plant-based is difficult (Graça et al., 2016), pointless and “not for me” (Oyserman, 2015). By rejecting claims against the consumption of animal products, omnivores may strengthen their commitment to eating animal products and their aversion for veg*nism (Bastian and Loughnan, 2017; Rothgerber, 2020). This individual-level polarization may spur group-polarization in society at large if omnivores publicly rationalize their diet and derogate those who oppose it, because in doing so, they may potentially recruit others to share and reinforce the carnist majority position (Kahan, 2013; Bastian and Loughnan, 2017). Omnivores with a weaker carnist identity (more likely less meat-attached or flexitarian, more liberal, less masculine and more feminine, higher solidarity for animals and less attached to cultural norms) are less likely to actively defend their diet; motivated ignorance and passive forms of veg*n stigmatization (e.g., avoidance) may suffice. These indirect defenses allow omnivores to ignore veg*n advocates’ claims, so that they remain ambivalent about the consumption of animal products (Rothgerber, 2020).

Overall, these findings are remarkably consistent with studies on minority influence (Moscovici, 1985; Mugny and Pérez, 1991; Martin et al., 2008; Levine and Tindale, 2014) showing that minority’s calls for change often evoke immediate defensiveness or only ambivalence. As a result, the influence minorities exert on majority members is usually non-existent or even negative *at a direct, manifest level* (Moscovici, 1985; Mugny and Pérez, 1991; Wood et al., 1994). This does not mean, however, that minorities exert no influence at all (Mugny and Pérez, 1991; Wood et al., 1994). In Western countries, the market of plant-based alternatives is growing and majority norms are gradually shifting as flexitarianism is gaining popularity and veg*nism is increasingly accepted (Vranken et al., 2014; Lundahl, 2020; Verain et al., 2022). Consequently, it may become increasingly difficult to defend animal-product consumption and advocates may become more influential (De Groeve and Rosenfeld, 2022). Both research on minority influence and cognitive dissonance suggests that omnivores may resolve moral/carnist identity threat by adopting a third dissonance-reducing strategy: committing to behavioral change.

### Commitment to behavioral change

A recurring pattern in studies that systematically compared minority versus majority influence is that minority (vs. majority) influence is characterized by changes that are private (rather than public) and indirectly (rather than directly) related to the position of the source (i.e., conversion), presumably because targets do not want to publicly align themselves with a stigmatized minority (Wood et al., 1994). This conversion can be immediate conversion, but is often delayed conversion.

#### Immediate conversion

Omnivores may (privately) accept claims that animal products entail avoidable harm (e.g., to animals, the environment) and might reduce dissonance by aligning their dietary behavior more with their moral identity and principles (Feinberg et al., 2019; Bouwman et al., 2022; De Groeve and Rosenfeld, 2022), thus rejecting animal-product consumption at least partially by making shifts toward veg*n diets (Rothgerber, 2020). One recent study (Silva Souza and O’Dwyer, 2022) found personal health arguments with a mixed recommendation (i.e., to reduce or eliminate animal products) could not persuade people to eat less animal products, but that arguments related to animal rights and environmental welfare were effective to increase omnivores’ willingness to reduce (not cease) animal-product consumption *via* elevated dissonance. In addition, participants exposed to environmental arguments were more likely to disagree with *ceasing* animal-product consumption. Arguably, health arguments do not consistently favor veg*n dieting (i.e., omnivorous diets can also be healthy), while environmental arguments appear less stringent and animal rights favor veg*n diets most consistently.

A meta-analysis of minority influence research suggests that consistency is especially important for minorities to exert influence (Wood et al., 1994). Not only does consistency allow to capture attention of the majority (Wood et al., 1994), it also allows to signal that the majority behaves inconsistently (Wrenn, 2018), should rethink their position and change their behavior to the minority position (Mugny and Pérez, 1991; Wood et al., 1994). Given that veganism is a consistent anti-speciesist position (Bruers, 2021), this might explain why animal-welfare interventions with a “go vegan” recommendation may have larger effects on meat reduction than more modest recommendations (“go vegetarian” or “reduce your consumption”) (Mathur et al., 2021). Similarly, Dakin et al. (2021) found that prescribing vegetarian (vs. flexitarian) diets for a week based on animal welfare arguments led to larger sustained reductions in meat intake, which was partially mediated by reduced 4Ns rationalization and commitment to eat meat. It is important to note, however, that participants in the studies above were probably already more receptive to eating less meat. As differences in opinion increase, a more flexible (vs. uncompromising) style of negotiation becomes more important for a consistent minority to exert influence (Mugny, 1975; Mugny and Pérez, 1991; Leenaert, 2017; Weiper and Vonk, 2021).

#### Delayed conversion

Minority influence research further suggests that conversion to a minority position is often delayed (rather than immediate) and typically happens after a validation process where majority members actively thought about the minority’s claims (Moscovici, 1980; Mugny and Pérez, 1991; Wood et al., 1994). Likewise, veg*ns typically report that they changed gradually in different stages (Chuck et al., 2016; Grassian, 2019; Bryant et al., 2022). Highlighting the role of motivated resistance, Bryant et al. (2022) provide an overview of psychosocial barriers to overcome in the journey to ethical veganism through five stages of change: precontemplation, contemplation, preparation, action, and maintenance. Consumers who reject rationalizations for eating animal products may become more ambivalent about eating meat and negative about conventional meat production systems (Berndsen and Van Der Pligt, 2004; Hartmann and Siegrist, 2020). Although they might initially be motivated to ignore claims against consuming animal products, they may be more open to eat meat alternatives (e.g., Quorn, tofu, seitan) (Hartmann and Siegrist, 2020) and change their diet after effortful information seeking if concerns about eating animal products can no longer be ignored (Rothgerber, 2020; Pauer et al., 2022). Based on interviews with veg*ns, this information may include a variety of sources, such as educational materials (e.g., documentaries, books, flyers, speeches), role models and emotionally intensive imagery related to animal cruelty (Chuck et al., 2016; Grassian, 2019).

Reducing carnist resistance seems crucial to promote dietary change among omnivores. As people reject carnism more, eat less meat, follow a flexitarian diet longer, and see avoiding meat as more self-defining, they are more likely to identify with vegetarians rather then meat-eaters (Rosenfeld et al., 2019) and less likely to negatively stereotype veg*ns as socially unattractive (Minson and Monin, 2012; De Groeve et al., 2022). A rejection of carnism is also strongly associated with more positive and less speciesist attitudes toward animals, feeling more guilty about eating animal products, and being more engaged in animal advocacy (Piazza et al., 2015; Monteiro et al., 2017; Rosenfeld, 2019b; Amiot et al., 2020). If moral reasons for veg*n diets are internalized, people are likely to develop disgust toward the idea of eating animal products (Rozin, 1996; Graça et al., 2016) and if eating veg*n diets feels identity-congruent, perceived difficulties in veg*n practice may be interpreted as worthwhile and meaningful (Oyserman, 2015). Nevertheless, important barriers for adopting veg*n diets (e.g., conformity, meat attachment, health concerns, practical convenience) may also cause a significant number of veg*ns to lapse or revert from veg*n diets temporarily or permanently (Rosenfeld, 2018; Salehi et al., 2020). Conversely, veg*ns are more likely to maintain their diet if they have social support, if they are motivated by animal ethics, if they have knowledge about veg*n nutrition and if it is practically feasible and affordable (Ruby, 2012; Salehi et al., 2020).

## Discussion

Having explained our theoretical account of carnist resistance to veg*n advocacy, we will now discuss directions for future research to test and qualify its main features (§3.1) (for a summary, see Table S1 in the Supplementary material). We further consider the need to go beyond veg*n advocacy (§3.2) and conclude (§3.3).

### Future research directions

#### Veg*n advocacy and moral identity

First, our account presupposes that veg*n advocacy is based on claims that animal-product consumption is harmful and avoidable. Future research could test to which extent veg*ns (vs. omnivores) perceive different non-veg*n diets and/or animal products as harmful (e.g., to animals, the environment, health) (e.g., Schein and Gray, 2015) and avoidable (e.g., by measuring “outcome efficacy”; Steg and de Groot, 2010). We expect that veg*ns are more likely to construe their diet as a part of their moral identity due to perceiving more avoidable animal harms, followed by environmental and health harms, respectively. More research is required to examine how veg*n dietary motivations (e.g., Hopwood et al., 2020, 2021b) contribute to moral identity internalization, as well as a desire to signal one’s moral identity (Aquino and Reed, 2002) *via* veg*n advocacy (e.g., Thomas et al., 2019; Judge et al., 2022).

#### Moral and carnist identity

Our account suggests that moral identity signaling among advocates may threaten omnivores’ moral and carnist identity simultaneously, and claims against animal-product consumption that are perceived as more harmful and avoidable are expected to arouse a stronger moral/carnist identity threat (e.g., by measuring “moral reproach”; Minson and Monin, 2012) and dissonant feelings (e.g., Silva Souza and O’Dwyer, 2022) among omnivores, especially among those with stronger moral and carnist identities. Future research could examine whether the strength of omnivores’ moral and carnist identity moderates threat perceptions of advocacy (see De Groeve and Rosenfeld, 2022) and also consider environmental and health harms related to various animal products as potential causes of dissonance besides animal harms related to meat in particular (Rothgerber, 2020; De Groeve and Rosenfeld, 2022; Silva Souza and O’Dwyer, 2022). Concerning moral identity, we acknowledge that people’s conceptions of what is “moral” may vary considerably, depending on various cooperative relationships (Curry et al., 2019), the endorsement of conventional values (i.e., authority, loyalty, purity) (Graham et al., 2013) and divine authority (Simpson et al., 2016). Although some research suggests that moral identity (Dawson et al., 2021) and moral judgment processes can be largely attributed to concerns about (intentional, unjustified) harms (Schein and Gray, 2015, 2018; Sousa et al., 2021), different moral paradigms may affect how people respond to veg*n advocacy (e.g., Grünhage and Reuter, 2021). Similarly, omnivores may vary considerably in how they construe their carnist identity, depending on the individual, culture, and the particular context in which it is cued (Turner and Reynolds, 2010; Oyserman, 2015). Pursuing a more comprehensive, multifaceted understanding of carnist identity, for example by conducting segmentation studies (e.g., Lacroix and Gifford, 2019; Verain et al., 2022), is recommended. Carnist resistance (i.e., pro-carnist and counter-veg*n defensiveness) is relevant to consider in this regard (see Table S2 for existing measurement scales).

#### Carnist resistance: Pro-carnist and counter-veg*n defenses

Based on our account, we expect that a stronger carnist identity positively predicts motivated reasoning, negative stereotyping of advocates, and commitment to eat animal products. More research could test whether motivated reasoning negatively predicts perceived harms and the perceived efficacy of veg*n diets to avoid harms, and whether different rationalizations for eating animal products (e.g., the 4Ns) support different negative stereotypes (e.g., veg*ns seen as contradicting the 4Ns). Conversely, omnivores with a weaker carnist identity should be less likely to rationalize animal-product consumption or actively stigmatize veg*ns by expressing negative stereotypes (though still more likely than veg*ns), and mainly rely on motivated ignorance. Researchers may examine whether different forms of motivated ignorance (e.g., ignoring farmed animal suffering) inform different stigmatizing attitudes (e.g., avoiding contact with veg*ns). Although relevant scales to measure stigmatization exist (Table S2), future research is needed to examine whether passive forms of stigmatization can be distinguished from negative stereotyping. We also recommend more psychometric analysis to better understand the interrelationship between pro-carnist defenses: for example, our conceptualization of neutralization as a form of motivated ignorance is rather tentative and the status of dichotomization is also less clear (Hartmann and Siegrist, 2020; Rothgerber, 2020). In addition, future research could assess the relative importance and interrelationship between personal and social motivations linked with one’s carnist identity related to individual meat attachment and healthy eating, politics (e.g., conservativism), gender (e.g., new/traditional masculinity), species (e.g., human supremacy) and culture (e.g., nation, religion) (see studies in §2.2.5 for measurement scales) and how these identities inform pro-carnist and counter-veg*n defenses. Lastly, research on how these defenses are associated with an ambivalence about or a commitment to animal-product consumption is recommended. For example, previous research has found both committed and ambivalent omnivores may be motivated to ignore/downplay the sentience of farmed animals (Buttlar and Walther, 2018; Leach et al., 2022), which might be due to differences in moral/carnist identity threat. Committed omnivores may ignore information due to indifference (i.e., low moral, high carnist identity threat), while ambivalent omnivores may want to avoid confrontation (i.e., higher moral identity threat) (Onwezen and van der Weele, 2016; Rothgerber, 2020), though this needs to be verified.

#### Commitment to behavioral change

Our account further suggests that an apparent resistance against veg*n advocacy may mask indirect, private influence, often at a later point in time. Therefore, future research on veg*n advocacy would benefit from integrating minority influence perspectives (Martin et al., 2008; Levine and Tindale, 2014), ideally using longitudinal designs to capture delayed influence across different stages of change (Bryant et al., 2022; De Groeve and Rosenfeld, 2022). More diverse quantitative and qualitative research approaches (e.g., field experiments, participant observation) are also recommended to demonstrate potential differences between publicly expressed and privately held beliefs (*cf.* pluralistic ignorance) (Bolderdijk and Cornelissen, 2022; De Groeve and Rosenfeld, 2022). In addition, our account suggests that the rejection of carnist beliefs is an important predictor of accepting commitments to dietary change (Rosenfeld et al., 2019; Trethewey and Jackson, 2019). Future research could thus develop interventions that target pro-carnist defenses, for example within an open, respectful dialogue (Buttlar et al., 2020). Although experimental studies have manipulated variables related to social norms and motivated ignorance such as dissociation and dichotomization (Mathur et al., 2021; Kwasny et al., 2022), experiments on how to tackle specific rationalizations (e.g., nice, natural, necessary, human supremacy, faith) and denial of harms are missing (Rothgerber, 2020; Kwasny et al., 2022). In addition, researchers could examine how to reduce negative stereotyping of vegan advocates, moralistic stereotyping in particular (for a review, see De Groeve and Rosenfeld, 2022).

Because our account suggests that promoting veg*n diets might increase polarization, we also recommend researchers to examine more pragmatic approaches to support change (De Groeve and Rosenfeld, 2022), for example by addressing the practical barriers (e.g., capacities, opportunities) that make changing one’s mind costly (Graça et al., 2019; Williams, 2020). For committed omnivores, the promotion of small dietary changes within meat formats that are already familiar (e.g., meat substitution) seems promising (Lacroix and Gifford, 2020). Emphasizing similarities between omnivores and veg*ns might also improve intergroup relations, trust and credibility (De Groeve and Rosenfeld, 2022), which could be examined using common and dual identity approaches (Gaertner et al., 1994; Dovidio et al., 2007). For example, to tackle human supremacy, advocates may emphasize commonalities (e.g., most people find factory farming problematic) and group differences (e.g., vegan diets minimize animal abuse) within a shared social identity (e.g., humans). To appeal to conservatives, veg*n advocates could argue that factory farming is untraditional and that environmental protection is patriotic (Rothgerber, 2020; Grünhage and Reuter, 2021). “Masculine” males may be motivated to challenge majority norms by emphasizing norms of responsibility, rebellion, and strength (Rothgerber, 2013). In addition, future research could examine the promotion of veg*n diets as a way to reclaim individuality: One does not have to identify with a particular group (veg*n or omnivore, male or female, liberal or conservative, etc.), in order to reflect on whether one’s diet violates one’s moral values (Bruers, 2021; Bouwman et al., 2022).

Although our account addresses resistance among omnivores against veg*n advocates, we also recommend future research to assess how motivated cognitions affect veg*ns’ commitment to their diets. Like omnivores, veg*ns may too eagerly embrace or suppress information that strengthens or protects their (moral) identity, for example by believing that humans are “naturally” herbivores, that non-veg*ns cannot care about animals or by denying that omnivorous diets can be healthy (van der Weele, 2013). Conversely, veg*ns may also comply with carnist norms in social situations if they experience stigma (Rosenfeld and Tomiyama, 2019; Bolderdijk and Cornelissen, 2022), for example by framing their diet as a requirement (e.g., allergies) rather than a (moral) choice or identity (Paxman, 2016; Rosenfeld and Tomiyama, 2019).

### Beyond veg*n advocacy

Lastly, given that there are many individual barriers for adopting plant-based diets, we also acknowledge the importance of institutional tactics to minimize harms of conventional animal-based diets, such as restructuring choice architecture (e.g., nudging, default-setting) (Bianchi, Garnett, et al., 2018b) and fiscal measures (i.e., taxes, subsidies) to dissuade animal-product consumption and promote the development of healthy, sustainable plant-based alternatives and cell-cultured meat, dairy and eggs (Grassian, 2019; Tubb and Seba, 2019; for a criticism of cell-cultured meat, see Chriki and Hocquette, 2020). “Less but better” animal products and production systems could also improve the global food system (Sahlin and Trewern, 2022), though “humane” narratives concealing inhumane treatment of animals to this day complicate the matter (Francione, 2020). While vegan principles may be reconcilable with regenerative, agroecological practices through veganic farming, it also remains questionable whether regenerative practices can function on any significant scale without functionalities of animals (e.g., manure) (see Weis & Ellis, 2021). In either case, we concur a radical rethinking of human-animal and environmental relationships is required (UNEP, 2020; Weis and Ellis, 2021) and momentum is growing to improve public and animal health systems (Cornish et al., 2016; UNEP, 2020), to urgently safeguard and restore terrestrial, marine and aerial wildlife habitats (Ripple et al., 2017; Stoll-Kleemann and Schmidt, 2017; Willett et al., 2019) and to legally recognize and protect farmed animals as sentient beings (Francione, 2020; Reese, 2020, 2021).

### Conclusion

Attempts to promote shifts toward veg*n diets are often met with resistance due to a variety of individual, social and contextual barriers (Graça et al., 2019). The present article integrates sociopsychological theorizing and empirical research to provide an account for omnivores’ ideological resistance to veg*n advocacy. We trace this “carnist resistance” back to a motivation among omnivores to avoid a salient threat to their moral and/or carnist identity. We theorized that pro-carnist defenses relying on motivated reasoning and ignorance inform negative stereotyping and stigmatization as counter-veg*n defenses. The maintenance of omnivores’ carnist identity can be personally motivated (i.e., meat attachment), but also socially motivated because of political, gender, species, and cultural identities associated with eating animal products. Meat-attached individuals, conservatives, men endorsing traditional masculinity and human supremacists are more likely to actively defend the consumption of animal products and negatively stereotype veg*ns. More ambivalent individuals (e.g., flexitarians), liberals, women and those with more solidarity for animals are less likely to rationalize animal-product consumption and actively stigmatize veg*ns; motivated ignorance and passive forms of stigmatization may suffice as defenses. An ideological resistance to veg*n advocacy reinforces commitment to and ambivalence about animal-product consumption, though attitudes toward animal products and veg*ns may vary across cultures. At the same time, there are signs that the zeitgeist in Western countries is shifting in favor of veg*n diets (Vranken et al., 2014; Verain et al., 2022), so veg*n advocates may become increasingly influential in inducing gradual behavioral change (De Groeve and Rosenfeld, 2022) *via* immediate or delayed conversion. Our account may inform scientists in developing testable hypotheses to gain understanding on how to remediate ideological resistance and may inform veg*n advocates in developing effective interventions for positive social change.

## Data availability statement

The original contributions presented in the study are included in the article/Supplementary material, further inquiries can be directed to the corresponding author.

## Author contributions

BDG developed the theoretical account and wrote the manuscript with BB and LH providing guidance and suggestions. All authors contributed to the article and approved the submitted version.

## Funding

This article elaborates on unpublished work performed during the doctoral research of BDG, which was supported by a PhD grant fundamental research of the Research Foundation - Flanders (FWO) (grant number FWO.3F0.2017.0033.01), though no funding from this source was used to produce this article.

## Conflict of interest

The authors declare that the research was conducted in the absence of any commercial or financial relationships that could be construed as a potential conflict of interest.

## Publisher’s note

All claims expressed in this article are solely those of the authors and do not necessarily represent those of their affiliated organizations, or those of the publisher, the editors and the reviewers. Any product that may be evaluated in this article, or claim that may be made by its manufacturer, is not guaranteed or endorsed by the publisher.
