# Supplementary material for: Ideological resistance to veg*n advocacy: An identity-based motivational account
Source: Front Psychol. 2022 Nov 30;13:996250. doi: 10.3389/fpsyg.2022.996250 (PMC9749860; doi:10.3389/fpsyg.2022.996250)
Supplement: Supplementary file 1 [file Data_Sheet_1.docx]

Supplementary Material

Table S1
*Future Research Questions and Recommendations*

| **Veg*n advocacy and moral identity** |
| --- |
| - To what extent do veg*ns (vs. omnivores) perceive different non-veg*n diets and/or animal products as harmful  (e.g., to animals, the environment, health) and avoidable? - How do veg*n dietary motivations contribute to moral identity internalization, moral identity signaling and  veg*n advocacy? |
| **Moral and carnist identity** |
| - Do claims against animal-product consumption that are perceived as more harmful and avoidable arouse stronger moral/carnist identity threat and dissonant feelings among omnivores? - Are omnivores’ threat perceptions moderated by the strength of their moral and carnist identity? - How does the type of animal product (e.g., meat vs. seafood vs. dairy vs. eggs, conventional vs. organic production systems) and the type of product-related harm (e.g., animal vs. environmental vs. health harms) affect dissonance and threat perceptions? - What is the role of different moral paradigms in perceiving and responding to veg*n advocacy? - How do omnivores construe their carnist identity? |
| **Carnist resistance: Pro-carnist and counter-veg*n defenses** |
| - Does a stronger (vs. weaker) carnist identity predict more (vs. less) motivated reasoning and negative stereotyping? - Does motivated reasoning negatively predict perceived harms and the perceived efficacy of veg*n diets to avoid harms? - Do different rationalizations for eating animal products predict different negative stereotypes of veg*n advocates? - Do different forms of motivated ignorance predict different stigmatizing attitudes? - What is the psychometric relationship between passive forms of stigmatization and negative stereotyping? - What is the psychometric interrelationship between different pro-carnist defenses? - How are neutralization and dichotomization psychometrically related to other defenses? - What is the relative importance and interrelationship between personal and social motivations linked with one’s carnist identity (e.g., meat attachment, health, politics, gender, species, culture)? - How do these personal and social identities inform pro-carnist and counter-veg*n defenses? - How are pro-carnist and counter-veg*n defenses associated with an ambivalence about or a commitment to  animal-product consumption? |
| **Commitment to behavioral change** |
| - Future research on veg*n advocacy should integrate minority influence perspectives. - Longitudinal designs may allow to capture delayed influence across different stages of change. - Future research should adopt more diverse quantitative and qualitative research approaches to capture differences between publicly expressed and privately held beliefs. - Which interventions are effective for reducing different forms of motivated ignorance and motivated reasoning? - Which interventions are effective for reducing negative (e.g., moralistic) stereotyping? - How can veg*n advocates avoid polarization? - Future research could examine practical barriers of veg*n diets that make changing one’s mind costly. - Future research could examine common and dual identity approaches to veg*n advocacy. - Future research could examine whether veg*n advocates are more effective if they appeal to values of their audience  (e.g., conservatives, traditional males). - Future research could examine whether veg*n diets can be promoted as a way to reclaim individuality. - Future research could examine how motivated cognitions affect veg*ns’ commitment to their diets. |

Table S2
*Existing Scales to Measure Pro-Carnist and Counter-Veg*n Defenses*

| **Pro-carnist defenses** | **Measurement scales** |
| --- | --- |
| Motivated reasoning | - the 4Ns can be measured using the 4Ns Scale (Piazza et al., 2015), or the novel Motivations to eat Meat Inventory (Hopwood et al., 2021a):   - Nice (e.g., “It is delicious”);  - Necessary (e.g., “It is necessary for good health”);  - Normal (e.g., “Everybody does it”);  - Natural (e.g., “Eating meat is part of our biology”) - Meat-Eating Justification subscales (Rothgerber, 2013):   - pro-meat justification (e.g., “I enjoy eating meat too much to ever give it up”);  - health justification (e.g., “We need meat for a healthy diet”);  - hierarchical justification (e.g., “Humans are at the top of the food chain and meant to eat   animals”);  - fate justification (e.g., “Our early ancestors ate meat, and we are supposed to also”);  - religious justification (e.g., “God intended for us to eat animals”);  - denial of animal pain (e.g., “Animals do not feel pain the same way humans do”) - slaughter justification (e.g., “It is okay to kill animals as long as it is done quickly and without pain”) (Hartmann & Siegrist, 2020)  - the Carnism Inventory (Monteiro et al., 2017) measures justifications for eating meat  (i.e., carnist defense, e.g., “Eating meat is better for my health”) and dominant attitudes toward animals (i.e., carnist dominance, e.g., “I have the right to kill any animal I want”) |
| Motivated ignorance | - self-reported willingness to ignore (e.g., “I ignore information regarding X”) (Onwezen & van der Weele, 2016)  - Meat-Eating Justification subscales (Rothgerber, 2013):   - avoidance (e.g., “I try not to think about what goes on in slaughterhouses”);  - dissociation (e.g., “I do not like to think about where the meat I eat comes from”);  - dichotomization (e.g., “I am more sensitive to the suffering of house pets like   cats and dogs than other wild animals”); - the compartmentalization of farm animals scale (e.g., “The animals that we eat are different from the other types of animals”) (Amiot et al., 2019) also measures dichotomization - perceived behavior change, or downplaying one’s current consumption can be measured using self-reported consumption after experimental manipulations (Rothgerber, 2014, 2020) - a scale to measure neutralization (e.g., claiming one behaves ethically enough or only eats animals that were treated or slaughtered humanely) is lacking (Dowsett et al., 2018) |
| **Counter-veg*n defenses** | **Measurement scales** |
| (Negative) stereotyping | - Minson and Monin (2012) used a bipolar scale to measure the evaluation of vegetarians  (e.g., kind–mean, stupid–intelligent, healthy–unhealthy, judgmental– nonjudgmental) - De Groeve et al. (2022) used unipolar scales to measure perceived moralistic traits  (e.g., arrogant, fanatical), sociability (e.g., friendly, sociable), perceived eccentricity (e.g., odd, unconventional), morality (e.g., ethical, kind-hearted), commitment  (e.g., dedicated, motivated), and healthiness (healthy, physically fit). |
| Stigmatization | - Vegaphobia (Vandermoere et al., 2019) measures negative and stigmatizing attitude towards veg*ns using four items (i.e., “I find people who do not eat meat and fish weird”; “When I cook for others, I do not take into account whether there are vegetarians among them. They have to eat whatever I make.”; “I wouldn’t like it if my children wanted to become vegetarians”; “Vegetarianism is a temporary trend that will blow over”) - the Vegetarianism Threat Scale (Dhont & Hodson, 2014) measures people’s perception of vegetarianism as a cultural threat (e.g., “The vegetarian movement is too involved in local and national politics.”)  - Attitudes Toward Vegetarianism Scale (Chin et al., 2002) measures perceived adequate treatment of vegetarians (e.g., “I avoid interacting with vegetarians whenever possible”, “It’s O.K. to tease someone for being a vegetarian”) as well as stereotypes about behaviors, beliefs and health/mental traits associated with vegetarians (e.g., “Vegetarians preach to much about their beliefs and habits”, “Vegetarians are overly concerned about animal rights”) |

*Note*. This list of existing measurement scales is not fully exhaustive.
